# Supplementary material for: Delivery of the VIVIT Peptide to Human Glioma Cells to Interfere with Calcineurin-NFAT Signaling
Source: Molecules. 2021 Aug 7;26(16):4785. doi: 10.3390/molecules26164785 (PMC8400789; doi:10.3390/molecules26164785)

Supplementary Materials

Delivery of the VIVIT peptide to human glioma cells to interfere with calcineurin-NFAT signaling

Aleksandra Ellert-Miklaszewska[1], Agata Szymczyk[1], Katarzyna Poleszak[1], Bozena Kaminska[1,*]

Figure S1. VIVIT peptide delivery scheme and expected mode of action.

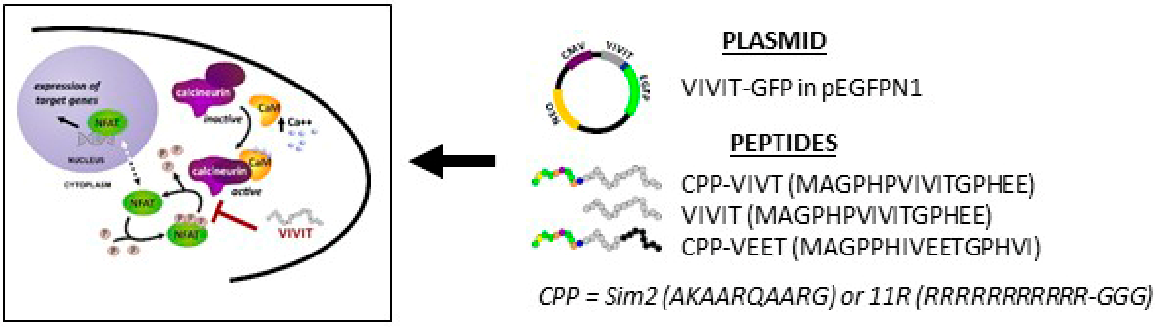

Figure S2. The effect of 11R-VIVIT and 11R-VEET peptides on the levels of NFAT genes expression. Gene expression was analyzed by qPCR in LN229 cells after 6 h treatment with the indicated concentrations of the peptides. Bars represent means ± SD from at least 3 experiments. Statistical significance of changes was determined using one-way ANOVA followed by Tukey post-hoc test (* p<0.05, ** p<0.01, ***p<0.001 vs UNT, # p<0.05, ## p<0.01, ### p<0.001 vs 11R-VEET).

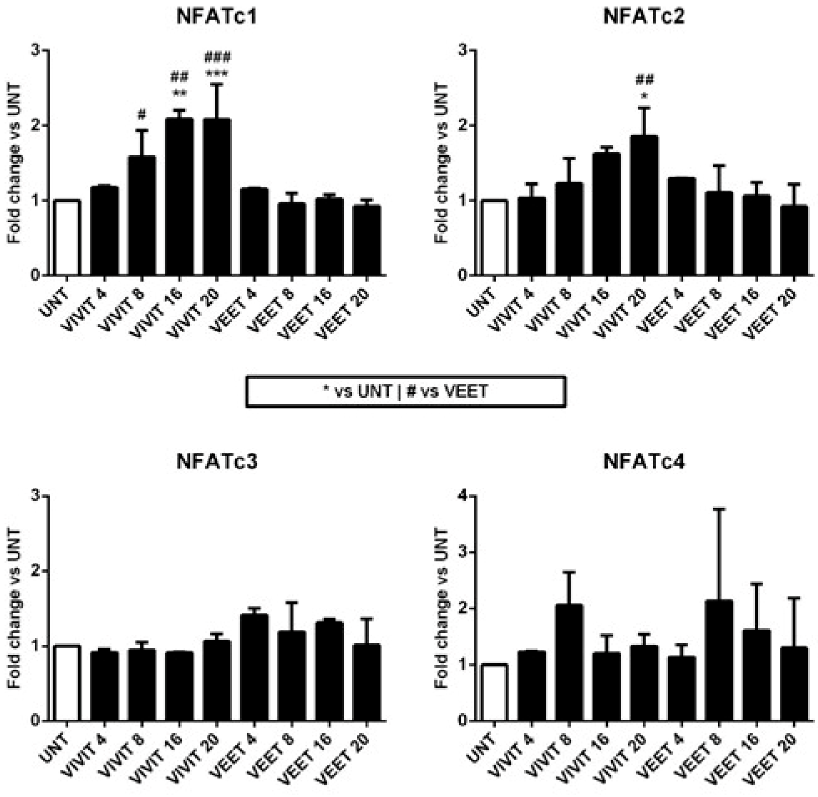

Figure S3. 11R-VIVIT enhances NFAT translocation to the nucleus. Distribution of NFATc3 in cytosolic and nuclear fractions was evaluated using Western blotting 4 h after treatment of U87 cells with 11R-VIVIT (20 μM) or CsA (5 μg/ml). GAPDH and Lamin B detection was used to assess the purity of the cytoplasmic and nuclear fractions, respectively.

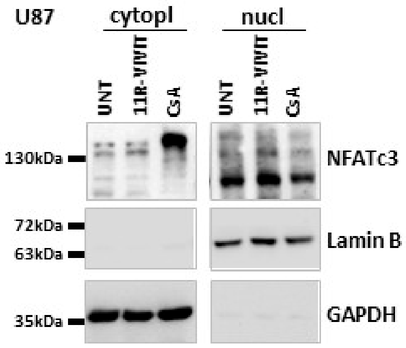

Supplement: Supplementary file 1 [file molecules-26-04785-s001.zip › molecules-1301481-supplementary.pdf]
